# D-GPM: A Deep Learning Method for Gene Promoter Methylation Inference

**DOI:** 10.3390/genes10100807

**Published:** 2019-10-14

**Authors:** Xingxin Pan, Biao Liu, Xingzhao Wen, Yulu Liu, Xiuqing Zhang, Shengbin Li, Shuaicheng Li

**Affiliations:** 1 BGI Education Center, University of Chinese Academy of Sciences, Shenzhen 518083, China; panxingxin16@mails.ucas.ac.cn (X.P.); biaoliu2019@gmail.com (B.L.); liuyulu@genomics.cn (Y.L.);; 2 School of Biological Science and Medical Engineering, Southeast University, Nanjing 210096, China; wenxingzhao1227@gmail.com; 3 College of Medicine and Forensics, Xi’an Jiaotong University, Xi’an 710061, China; 4 Department of Computer Science, City University of Hong Kong, Kowloon 999077, Hong Kong

**Keywords:** promoter methylation, deep neural network, machine learning, landmark genes, target genes

## Abstract

Whole-genome bisulfite sequencing generates a comprehensive profiling of the gene methylation levels, but is limited by a high cost. Recent studies have partitioned the genes into landmark genes and target genes and suggested that the landmark gene expression levels capture adequate information to reconstruct the target gene expression levels. This inspired us to propose that the methylation level of the promoters in landmark genes might be adequate to reconstruct the promoter methylation level of target genes, which would eventually reduce the cost of promoter methylation profiling. Here, we propose a deep learning model called Deep-Gene Promoter Methylation (D-GPM) to predict the whole-genome promoter methylation level based on the promoter methylation profile of the landmark genes from The Cancer Genome Atlas (TCGA). D-GPM-15%-7000 × 5, the optimal architecture of D-GPM, acquires the least overall mean absolute error (MAE) and the highest overall Pearson correlation coefficient (PCC), with values of 0.0329 and 0.8186, respectively, when testing data. Additionally, the D-GPM outperforms the regression tree (RT), linear regression (LR), and the support vector machine (SVM) in 95.66%, 92.65%, and 85.49% of the target genes by virtue of its relatively lower MAE and in 98.25%, 91.00%, and 81.56% of the target genes based on its relatively higher PCC, respectively. More importantly, the D-GPM predominates in predicting 79.86% and 78.34% of the target genes according to the model distribution of the least MAE and the highest PCC, respectively.

## 1. Introduction

By influencing the DNA accessibility, methylation of the promoter of a gene regulates various biological processes, including gene expression, imprinting regulation, cell differentiation, X chromosome inactivation, and tissue-specific gene regulation [1,2,3,4]. Several experimental methods have been gradually developed for profiling DNA methylation, and these methods can be broadly grouped into protocols based on enzymatic digestion, affinity enrichment, and bisulfite conversion [5,6,7,8]. Despite technological advances, there are still limitations in these existing wet-lab methods. Protocols based on enzymatic digestion leverage methylation-sensitive restriction enzymes, which have differential digestion properties for methylated and unmethylated CpG sites. Although enzymatic digestion-based approaches enable genome-wide methylation profiling and are cost-effective, the resolution of these approaches is restricted to regions adjacent to the methylation-sensitive restriction enzyme recognition sites, and they cannot quantify the methylation level of single CpG sites [9]. Affinity enrichment-based protocols enrich for methylated DNA fragments by either methyl-binding domain proteins or antibodies. Methylated DNA immunoprecipitation uses anti-methylcytosine antibodies to bind and quantify 5mC, but the resolution of this method is limited to 100–300 base pair-long fragments, and it is also biased towards hypermethylated regions [10]. Illumina’s 450 K bead-chip is the most widely used bisulfite microarray for profiling DNA methylation in humans, and two distinct primers are used to distinguish between methylated and unmethylated fragments. However, the chip only probes approximately 450 K CpG sites in the human genome, covers partial CpG islands, and may be biased towards CpG-dense contexts [11]. Whole-genome bisulfite sequencing detects C → T conversions by sequencing bisulfite-treated fragments and aligning the sequenced fragments back to a reference genome, and it is considered as a golden standard protocol since it can profile DNA methylation at a single cytosine resolution genome-wide. However, it is too costly because the genome-wide deep sequencing of bisulfite-treated fragments needs to generate a compendium of the gene methylation level over a large number of conditions, such as in the context of a retrovirus, DNA methyltransferase activity changes, and drug treatments [12]. Currently, the community is awaiting more feasible and economical solutions.

Previous research suggests that there are a large number of genes across the whole human genome, but most of their expression profiles are considered to be highly correlated, i.e., by leveraging the inner correlation between genes, the expression level of a few well-chosen landmark genes captures sufficient detail to reconstruct the expression of the rest of genes, namely, the target genes across the genome [13]. The above result was achieved by studying the gene regulation networks and conducting a principal component analysis of the whole-genome expression profile from the CMap data [14]. Motivated by these findings, scientists have developed a new technology called the L1000 Luminex bead, which only acquires the expression profiling of the landmark genes (∼1000) to infer the expression profiling of the target genes (∼21,000) [15].

Inspired by L1000, we proposed a method to infer the promoter methylation of the target genes according to the promoter methylation of the landmark genes, thus acquiring the whole-genome promoter methylation level and characterizing the cellular states of samples under various conditions, with a much lower cost. The rationale is as follows: first, latent associations exist between the expression of these landmark genes and the target genes at the genome-wide level [13]; second, methylation in the promoters located upstream of the transcription start site is usually negatively correlated with their corresponding gene expression levels [16]. Hence, it is likely that strong associations are present among the promoter methylation levels in the landmark genes and target genes, and computationally inferring the promoter methylation of target genes based on landmark genes is theoretically feasible.

To predict the methylation panorama of the whole genome is a large-scale multitask machine learning problem, with a high-dimensional aim (∼21,000) and a low-dimensional attribute (∼1000). Meanwhile, the deep learning method has shown its superior power in integrating large-scale data and capturing the nonlinear complexity of input features over the years. In biology, extensive applications of deep learning methods include predictions of the splicing activity of individual exons, inferring chromatin marks from the DNA sequence, and quantification of the effect of single nucleotide variants on chromatin accessibility [17,18,19].

Here, we present a multilayer neural network named Deep-Gene Promoter Methylation (D-GPM) to tackle the above large-scale multitask problem. To evaluate our D-GPM model, we benchmarked its performance against linear regression (LR), the regression tree (RT), and the support vector machine (SVM), with regard to methylation profile data based on the Illumina Human Methylation 450 k data from The Cancer Genome Atlas (TCGA) [20]. LR can be used to infer the promoter methylation of the target genes based on the promoter methylation of the landmark genes by training linear regression models independently for each target gene in methylation data. However, the linear model may fail to capture the nonlinear relations of the original data. The SVM reliably represents complex nonlinear patterns, but is limited by its poor scalability due to the large amount of data. The RT is beneficial due to its ability to increase the interpretability of the biological data and prediction model, despite its lower accuracy and instability in some predictors.

**Figure 1:**
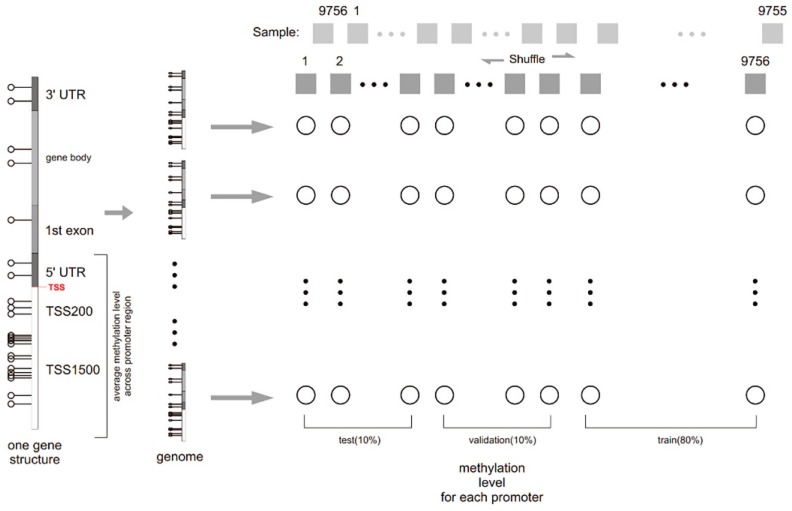
The workflow for training promoter methylation prediction models. After accessing location information on the promoter region of all genes, namely the 1500 bp from upstream of the TSS site (5′end) to 500 bp downstream (3′end) range, we determined the relationship between probes and gene promoter regions. The mean β value calculation of all the probes located in the promoter region of a certain gene is defined as its promoter methylation level. The methylation data was randomly partitioned into 80% for training, 10% for validation, and 10% for testing.

According to Illumina Human Methylation 450 k data, we accessed the promoter methylation information about 902 landmark genes and 21,645 target genes [21]. The promoter region was defined as from 1.5 kb upstream to 0.5 kb downstream of the RefSeq transcription start sites, according to MethHC, as shown in Figure 1 [22]. The experimental results show that D-GPM consistently outperforms the three methods for the data tested, and this was measured using the criteria of the mean absolute error (MAE) and the Pearson correlation coefficient (PCC).

## 2. Materials and Methods

In this section, we first specify the gene methylation datasets used in this study and formulate the gene promoter methylation inference problem. We then propose the D-GPM for this problem and describe the relevant details. Finally, we introduce three machine learning methods, which serve as benchmarks.

### 2.1. Datasets

The methylation β value (MBV) datasets were acquired from TCGA [20]. Considering that the Illumina Human Methylation 450 k possesses more probes and a higher coverage rate, we excluded datasets from the Illumina Human Methylation 27 k, and finally, 9756 records remained for the analysis [21]. After filtering out the records, we calculated the average β value of all the probes located in the promoter regions of a certain gene as its corresponding promoter methylation level, according to MethHC [22].

We randomly partitioned the methylation data into 80% for training, 10% for validation, and 10% for testing, which corresponded to 7549 samples, 943 samples, and 943 samples, respectively. We denoted them as MBV-tr, MBV-va, and MBV-te, respectively. MBV-va was utilized in various processes, including model selection and hyperparameter tuning.

### 2.2. Multitask Regression Model for Gene Expression Inference

In the model, there are *J* landmark genes, *K* target genes, and *N* training samples. We denoted the training data as ${\left\{x_{i},y_{i}\right\}}_{i=1}^{N}$, where $x_{i}\in {ℜ}^{J}$ represents the promoter methylation profiles of the landmark genes and $y_{i}\in {ℜ}^{K}$ represents the methylation profiles of the target genes in the *i*th sample. Our task was to find a mapping $F:{ℜ}^{J}\Rightarrow {ℜ}^{K}$ that fits ${\left\{x_{i},y_{i}\right\}}_{i=1}^{N}$ well, which can be viewed as a multitask regression problem.

As for the multitask regression task, let us assume a sample of *N* (*N* = 9756 samples) individuals, each represented by a *J*-dimensional input vector (*J* = 902 landmark genes) and a *K*-dimensional output vector (*K* = 21,645 target genes). Let ***X*** denote the N×J input matrix, whose column corresponds to the observations for the *j*-th input $x_{j}=\{x_{j}^{1},\ldots ,{x_{j}^{N}\}}^{T}$. Let ***Y*** denote the N×K output matrix, whose column is a vector of observations for the *k*-th output $y_{k}=\{y_{k}^{1},\ldots ,{y_{k}^{N}\}}^{T}$. For each of the *K* output variables, we assume a linear regression model:

(1) $$y_{k}=X\beta_{k}+\varepsilon_{k},\forall k=1,\ldots K,$$

where $\beta_{k}$ is a vector of the *J* regression coefficients $\{\beta_{k}^{1},\ldots {\beta_{k}^{J}\}}^{T}$ for the *k*-th output, and $\varepsilon_{k}$ is a vector of *N* independent error terms having a mean of 0 and a constant variance. We centered the $y_{k}{}^{,}s$ and $x_{j}{}^{,}s$ such that $\sum_{i}y_{k}^{i}=0$ and $\sum_{i}x_{j}^{i}=0$, and considered the model without an intercept.

The regression coefficients matrix $\beta_{}$ was used to take advantage of the relatedness across all the input variables.

### 2.3. Assessment Criteria

We adopted MAE and PCC as the criteria to evaluate the models’ performance at each target gene *t* of the different samples. We formulated the overall error as the average MAE over all the target genes. The PCC was used to describe the relationship between the real promoter methylation and the predicted promoter methylation of each target gene. Here, the definitions of MAE and PCC for evaluating the predictive performance at each target gene *t* are as follows:

(2) $$MAE_{\left(t\right)}=\frac{1}{N^{’}}\sum_{i=1}^{N’}|y_{i(t)}-{\hat{y}}_{i(t)}|,$$

(3) $$Correlation_{\left(t\right)}=\frac{\sum_{i=1}^{N^{’}}\left(y_{i(t)}-{\bar{y}}_{\left(t\right)})({\hat{y}}_{i(t)}-{\bar{\hat{y}}}_{\left(t\right)}\right)}{\sqrt{\sum_{i=1}^{N^{’}}{\left(y_{i(t)}-{\bar{y}}_{\left(t\right)}\right)}^{2}\cdot \sum_{i=1}^{N^{’}}{\left({\hat{y}}_{i(t)}-{\bar{\hat{y}}}_{\left(t\right)}\right)}^{2}}},$$

where *N’* is the number of samples tested; ${\hat{y}}_{i\left(t\right)}$ is the predicted expression value for the target gene *t* in sample *i*; and ${\bar{\hat{y}}}_{\left(t\right)}$ is the mean predicted expression value for the target gene *t* in *N’* testing samples.

### 2.4. D-GPM

D-GPM is a fully connected multilayer perceptron with one output layer. All the hidden layers consist of *H* hidden units. In this work, we employed a set of *H*s, ranging from 1000 to 9000, with a step size of 1000. A hidden unit *j* in layer *l* takes the sum of the weighted outputs plus the bias from the previous layer *l* − 1 as the input and produces a single output $o_{j}^{l}$:

(4) $$o_{j}^{l}=f\left(\sum_{i=1}^{H}w_{i,j}^{l-1}o_{i}^{l-1}+b_{j}^{l-1}\right),$$

where *H* is the number of hidden units; ${\left\{w_{i,j}^{l-1},b_{j}^{l-1}\right\}}_{i=1}^{H}$ represents the weights and the bias of unit *j* to be found; and f is a nonlinear activation function named *Tanh*, which is

(5) $$f\left(x\right)=\frac{e^{x}-e^{-x}}{e^{x}+e^{-x}},.$$

The loss function is the sum of the mean squared error at each output unit, which is

(6) $$\varsigma =\sum_{t=1}^{T}\left[\frac{1}{N}{\sum_{i=1}^{N}\left(y_{i(t)}-{\overset{\wedge }{y}}_{i(t)}\right)}^{2}\right].$$

**Figure 2:**
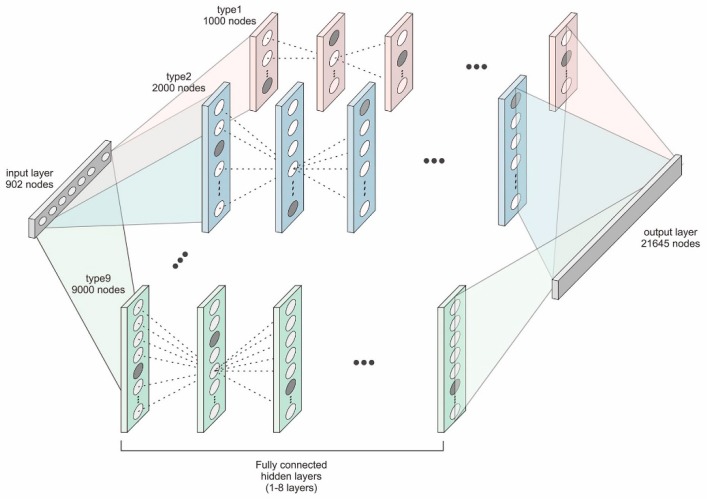
The architecture of Deep-Gene Promoter Methylation (D-GPM). It is comprised of one input layer, one or multiple hidden layers, and one output layer. All the hidden layers have the same number of hidden units.

D-GPM contained 902 units in the input layer, corresponding to the 902 landmark genes, and we also configured D-GPM with 21,645 units in the output layer analogous to the 21,645 target genes. Figure 2 shows the various architectures of D-GPM.

Here, we briefly describe the training techniques introduced into D-GPM and their significance in training steps:

1. Dropout is a scheme used to perform model averaging and regularization for deep neural networks [23]. Here, we utilize dropout for all the hidden layers of D-GPM, and the dropout rate p, which steers the regularization intensity, is set at [from 0% to 50%, with step size 5%], separately, to find the optimum architecture of D-GPM;
2. Normalized initialization can stabilize the variances of activation during epochs [24]. To initialize the parameters of deep neural networks, here, we set the initialized weights to within the range of $\left[-1\times 10^{-4},1\times 10^{-4}\right]$, according to the activation function;
3. The momentum method is also adopted in our work to speed up the gradient optimization and improve the convergence rate of the deep neural networks [24];
4. The learning rate is initialized to $5\times 10^{-4}$, $2\times 10^{-4}$, $1\times 10^{-4}$, or $8\times 10^{-5}$, depending on the different architecture of D-GPM, and is tuned according to the training error on a subset of MBV-tr;
5. Model selection is implemented based on the MBV-va. The models are assessed on MBV-va after each epoch, and the model with the minimum loss function is saved. The maximum epoch for training is set as 500 epochs.

Here, we implement D-GPM with the Theano and Pylearn2 libraries [25,26].

### 2.5. Benchmark Methods

To evaluate the performance of the deep learning methods, we adopted LR, RT, and SVM as benchmarks.

We utilized the RT models with the rpart package with parameter testing (complexity: from 0.005 to 0.1, with step size 0.005) [27]. When the complexity parameter is 0.03, the RT model obtains the least MAE for MBV-te. The Gaussian RBF kernel function has a high superiority for a large sample and for high dimensional data, and it reduces the computational complexity in our methylation profiling data efficiently [28]. We adopted the kernlab package to implement SVM for predicting promoter methylation with parameter testing (cost of constraints violation: from 0.1 to 5, with step size 0.1) [29]. When the cost is 2.3, the SVM model obtains the least MAE for MBV-te. As for the linear model, in addition to a simple linear regression model, we introduced L1 or L2 penalties for regularization purposes. The simple linear regression model without any penalty outperforms linear regression models with L1 or L2 penalties on MBV-te concerning the overall MAE. This is probably because the linear model fails to capture intrinsic characteristics between promoter methylation of the landmark genes and target genes, resulting in underfitting. Therefore, regularization methods do not help in reducing the overall MAE for MBV-te.

## 3. Results

Here, we introduced the methylation β value (MBV) datasets from TCGA and defined the methylation profile inferences as a multitask regression problem, with the MBV-tr for training, MBV-va for validation, and MBV-te for testing. We have also illustrated our deep learning method D-PGM and the other three methods, including LR, RT, and SVM, to work out the regression problem. Next, we show the predictive performances of the above methods for the MBV-te data based on the MAE and PCC criteria.

### 3.1. D-GPM Performs the Best for Predicting Promoter Methylation

The back-propagation algorithm, mini-batch gradient descent, and other beneficial deep learning techniques are adopted in training the D-GPM [30]. The detailed parameter configurations are shown in Table 1.

According to the parameter configurations, all the combinations of parameters are made during the D-GPM training for predicting the promoter methylation of the target genes.

As Table 2 indicates, D-GPM acquires the best MAE performance for MBV-te, with five hidden layers of 7000 units and a 15% dropout rate (D-GPM-15%-7000 × 5) among the 792 (8 × 9 × 11) various D-GPMs. Meanwhile, D-GPM has an extraordinary edge over MAE compared with LR, SVM, and RT.

Similarly, D-GPM-15%-7000 × 5 also obtains the best PCC performance for MBV-te among the 792 prediction models, as shown in Table 3. The complete MAE and PCC evaluation of D-GPM armed with all the other architectures (hidden layer: from 1 to 8, with step size 1; hidden unit: from 1000 to 9000, with step size 1000; dropout rate: from 0% to 50%, with step size 5%) for the MBV-te is given in the Supplementary Materials.

Based on the above overall MAE and PCC performance for all the target genes, we can conclude that D-GPM, with five hidden layers of 7000 units and a 15% dropout rate, is the best model for predicting promoter methylation among the prediction models.

### 3.2. Evaluation According to MAE Criteria

**Figure 3:**
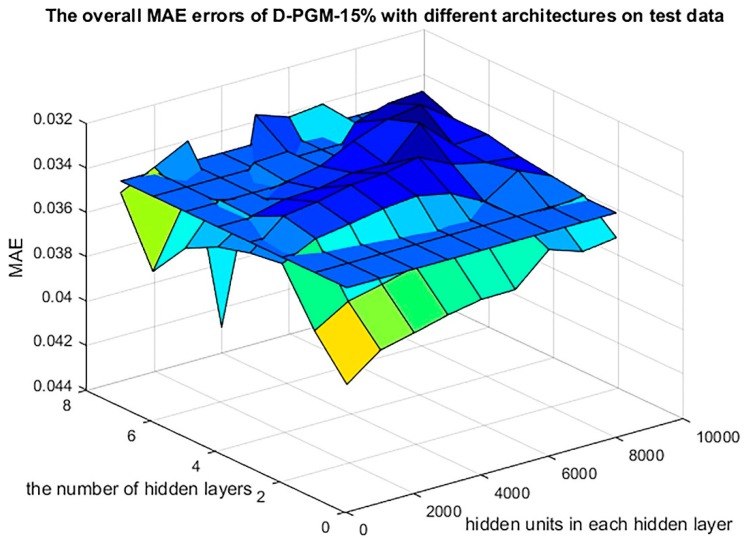
The overall MAE errors of D-GPM-15%, with various architectures for the MBV-te. The overall MAE errors of SVM for the MBV-te are shown as a cross section, which is 0.0350 high in terms of the MAE and serves as a benchmark to evaluate the MAE of D-GPM-15% with various architectures.

D-GPM acquires the best overall MAE performance for the MBV-te, with a 15% dropout rate (described as D-GPM-15%) among the eleven dropout rates, ranging from 0% to 50%, with a step size of 5%. Figure 3 shows the overall MAE performances of D-GPM-15% for the MBV-te and SVM acts as a benchmark. The larger architecture of D-GPM-15% (five hidden layers with 7000 hidden units in each hidden layer, described as D-GPM-15%-7000 × 5) acquires the least MAE for the MBV-te. D-GPM-15%-7000 × 5 outperforms LR by reducing the MAE by 9.59%, RT by reducing the MAE by 27.58% and SVM by reducing the MAE by 6.14%, respectively. A possible explanation for this is that deep learning models possess much richer representability, capture complex features, and make learning intrinsic characteristics much easier [31].

**Figure 4:**
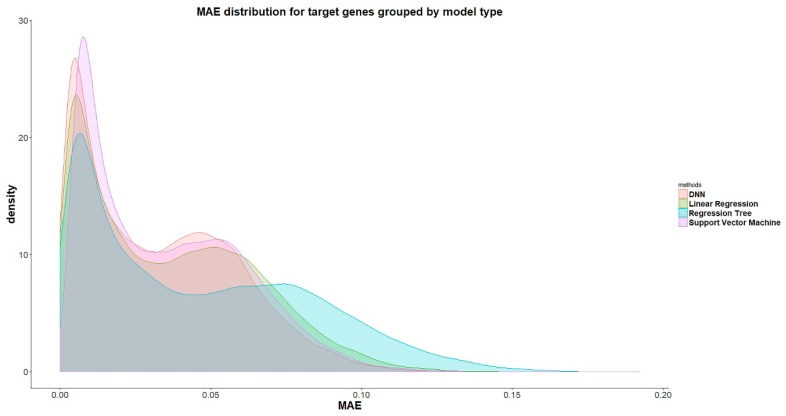
The density plots of the MAE by LR, RT, SVM, and D-GPM for the MBV-te.

D-GPM also outperforms LR and RT for almost all the target genes in terms of the MAE. Figure 4 shows the density plots of the MAE of all the target genes by LR, RT, SVM, and D-GPM. On the whole, D-GPM occupies a larger proportion at the low MAE level and a lower proportion at the high MAE level compared to the three machine learning methods, especially for RT and LR, attesting to the prominent performance of D-GPM.

**Figure 5:**
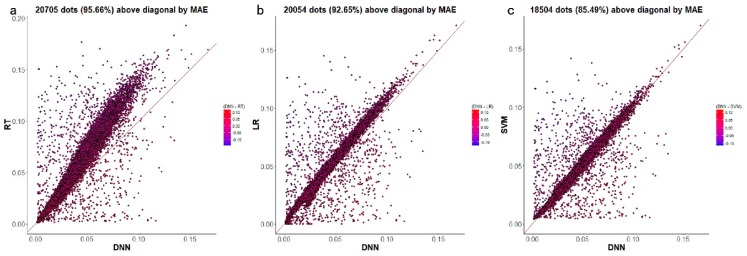
The predictive MAE of each target gene by D-GPM compared with RT, LR, and SVM for MBV-te. Each dot represents one of the 21,645 target genes. The x-axis is the MAE of each target gene obtained by D-GPM, and the y-axis is the MAE of each target gene obtained by the other machine learning method. The dots above the diagonal indicate that D-GPM achieves lower MAE compared with the other method. (**a**) D-GPM versus RT. (**b**) D-GPM versus LR. (**c**) D-GPM versus SVM.

Figure 5a–c displays a gene-wise comparative analysis of D-GPM and the other three methods. In terms of MAE, D-GPM outperforms RT for 95.66% (20,705 genes) of the target genes, outperforms LR for 92.65% (20,054 genes) of the target genes, and outperforms SVM for 85.49% (18,504 genes) of the target genes. These results can also be viewed by the larger proportion of dots that lie above the diagonal, and the better performance may suggest that D-GPM can capture some intrinsic nonlinear features of the MBV data, which LR, RT, and SVM did not accomplish.

RT performs significantly worse than the other methods in terms of the MAE. One possible reason for this is that the model is too oversimplified to capture the essential features between promoter methylation of the landmark and target genes based on the MBV-te [32].

**Figure 6:**
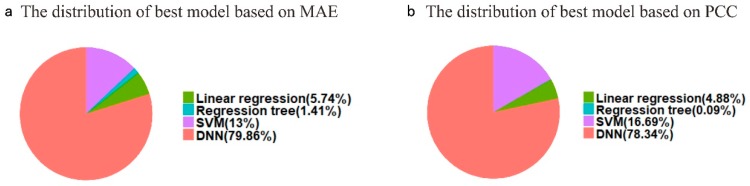
Distribution of the best model. (**a**) Distribution of the best model according to MAE for the target genes. (**b**) Distribution of best model according to PCC for the target genes.

According to the model distribution of the lowest MAE for each target gene, we found the best model distribution, as shown in Figure 6a. RT accomplishes the best MAE performance for 305 target genes (1.41%), including the genes *BRD2*, *GPI*, *MAF*, and *MICB*, implying that there is a relatively simple promoter methylation regulation mechanism and promoter methylation of these target genes may be dominantly regulated by a very few specific landmark genes. The LR can predict 1242 target genes (5.74%), at best, among the other three methods, including the genes *ABCD1*, *HPD*, *AMH*, and *ARAF*, laying a solid foundation for the pathogenesis of diseases, such as Adrenoleukodystrophy, Hawkinsinuria, Persistent Mullerian Duct Syndrome, and Pallister-Killian Syndrome, using our LR [33,34,35,36]. Noticeably, SVM performs best for a total of 2813 genes (13.00%), including *ACE2*, *A2M*, and *CA1*. One possible explanation for this is that there seem to be intricate and complicated interactions among the promoter methylation of the landmark genes and these 2813 target genes. Undoubtedly, D-GPM does better than the other three methods, as far as 17,285 target genes (79.86%) are concerned, demonstrating the deep neural networks’ powerful ability to capture the nonlinear relationship of methylation profiling.

### 3.3. Evaluation According to PCC Criteria

**Figure 7:**
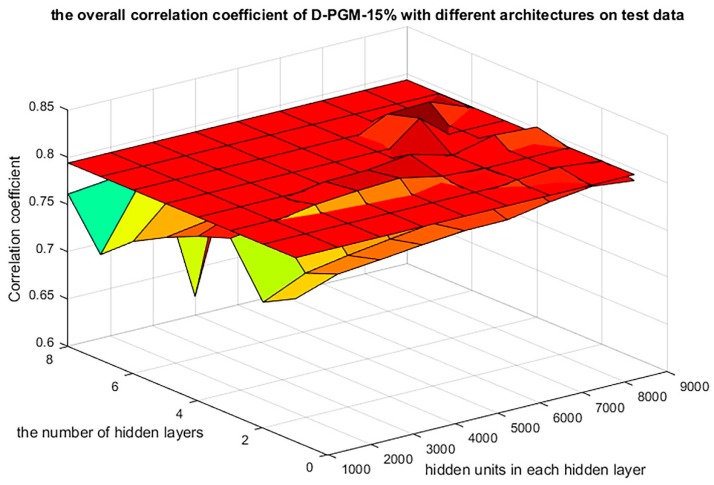
The overall PCC performance of D-GPM-15% with various architectures for the MBV-te. The overall PCC performance of SVM for the MBV-te is shown as a cross section, which is 0.794183 high in terms of the PCC and serves as a benchmark to evaluate the PCC of D-GPM-15% with various architectures.

D-GPM accomplishes the best overall PCC performance for the MBV-te, with a 15% dropout rate. Figure 7 shows the overall PCC performances of D-GPM-15% and the other methods for MBV-te. Similar to the MAE, D-GPM-15%-7000 × 5 acquires the most significant PCC for the MBV-te. The relative PCC improvement of D-GPM-15%-7000 × 5 is 4.34% compared to the LR, 22.96% compared to RT, and 3.07% compared to SVM. Similar to the MAE, almost all the combined architectures of D-GPM-15% outperform LR and RT in terms of the PCC performance.

**Figure 8:**
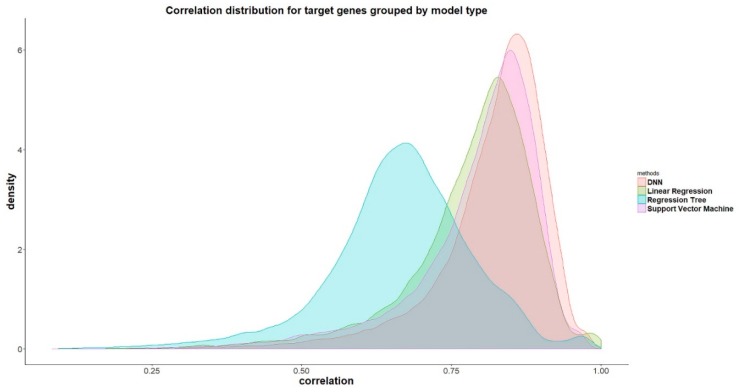
The density plots of the PCC of all the target genes by LR, RT, SVM, and D-GPM for MBV-te.

In terms of the PCC, D-GPM also outperforms RT and LR for almost all the target genes. Figure 8 displays the density plots of the PCC of all the target genes by LR, RT, SVM, and D-GPM. By and large, we can see that D-GPM possesses a larger proportion at the high PCC level and a lower proportion at the low PCC level compared to the RT, LR, and SVM.

**Figure 9:**
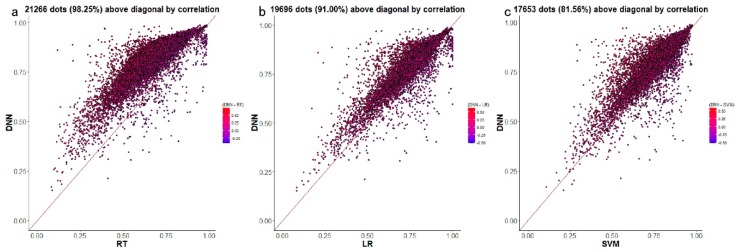
The predictive PCC of each target gene by D-GPM compared with RT, LR, and SVM for MBV-te. Each dot represents one out of the 21,645 target genes. The x-axis is the PCC of each target gene obtained by the above-mentioned three machine learning techniques, and the y-axis is the PCC of each target gene obtained by D-GPM. The dots above the diagonal indicate that D-GPM achieves a higher PCC value compared with the other method. (**a**) D-GPM versus RT. (**b**) D-GPM versus LR. (**c**) D-GPM versus SVM.

Figure 9a–c shows a gene-wise comparative analysis of D-GPM and the other three methods. For PCC, D-GPM outperforms RT in 98.25% (21,266 genes) of the target genes, LR in 91.00% (19,696 genes) of the target genes, and SVM in 81.56% (17,653 genes) of the target genes. Therefore, D-GPM’s powerful predictive performance for the PCC of all the target genes is preserved, similar to its effective prediction for the MAE. It is obvious that although the prediction property of the SVM is still modest in terms of the PCC, its predictive PCC performance for a number of the target genes is significantly higher than D-GPM. This finding is probably due to the fact that SVM is based on the principle of structural risk minimization, avoiding overlearning problems, and having a strong generalization ability.

According to the model distribution of the maximal PCC for each target gene, we found the best model distribution, as shown in Figure 6b. Surprisingly, RT only obtains the best PCC performance for 19 target genes (0.09%), including the genes *ALG1* and *NBR2*, falling far below the best 305 target genes in terms of the MAE. Considering RT’s awful predictive power for the PCC performance compared with its better predictive power for the MAE performance, this may be explained by the fact that RT makes decisions based on an oversimple assumption. LR predicts the best 1057 target genes (4.88%) among the other three methods, including the genes *AASS* and *ACE*, and the proportion of the best target genes of LR in the PCC level is similar to that in the MAE level. For the PCC, SVM is on its best behavior for a total of 3613 target genes (16.69%), having an increasing number compared to that of the MAE, in contrast to RT. Undoubtedly, D-GPM outperforms the other three methods, with regard to 16,956 genes (78.34%), in terms of the PCC.

**Figure 10:**
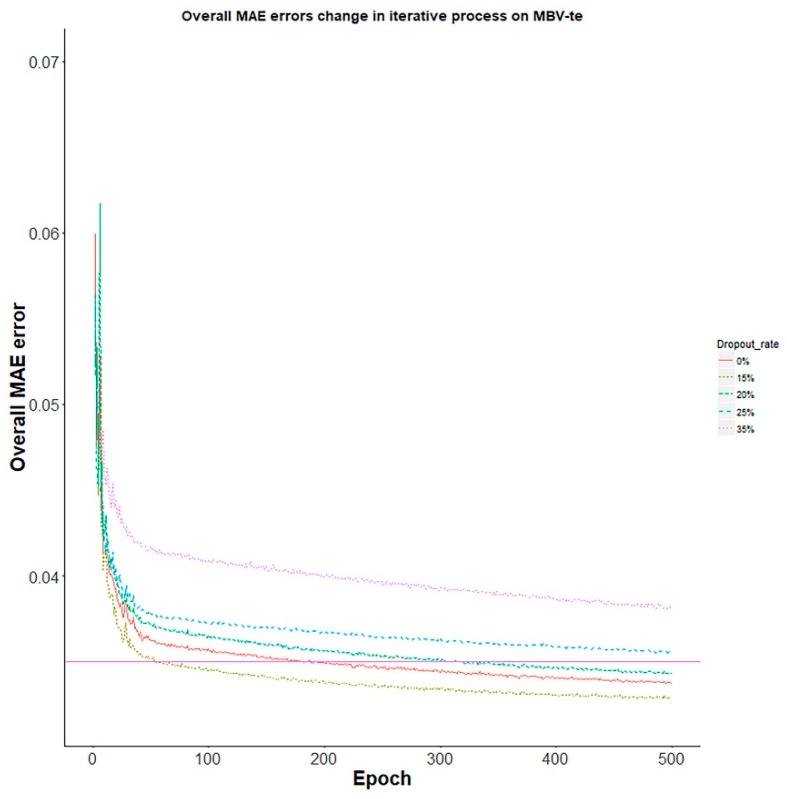
The overall MAE decreasing curves of D-GPM-15%-7000 × 5 for the MBV-te, with different dropout rates. The x-axis is the training epoch and the y-axis is the overall MAE error. The overall MAE error of SVM is also covered for comparison.

Noticeably, the dropout regularization manages to improve the performance of D-GPM-15%-7000 × 5 for the MBV-te, as shown in Figure 10. With a 15% dropout rate, D-GPM-15%-7000 × 5 consistently achieves the best MAE performance for the MBV-te among the models, with 0%, 15%, 20%, 25%, and 35% dropout rates.

## 4. Discussion

Comprehending intricate regulation modes of promoter methylation profiling under numerous biological states requires robust inference frameworks and cost-effective profiling tools. Despite the fact that whole-genome bisulfite sequencing is thought of as the best protocol, it is too costly because genome-wide deep sequencing of the bisulfite-treated fragments needs to be implemented. Promoter methylation of a gene has been confirmed to be associated with DNA accessibility and the binding of transcription factors that can regulate gene expression. Considering that there is an underlying relationship between ∼1000 landmark genes and ∼21,000 target genes at the genome-wide level of expression and the fact that methylation occurring in the promoters located in the promoter region is negatively associated with the expression of its corresponding gene, we can make good use of the methylation levels in the promoter regions of landmark genes to characterize the cellular status of samples under diverse conditions. Here, we also used three machine learning methods, namely, LR, SVM, and RT, and a deep method called D-GPM, for inferring the promoter methylation of target genes based on only landmark genes. The different methods have different advantages and disadvantages. RT provides us with a good interpretability, but exhibits a relatively lousy accuracy. LR offers a better performance compared to RT, even though it ignores the nonlinearity within biological data. SVM represents complex nonlinear patterns. Unfortunately, it suffers from a poor scalability of the large amount of data. To some extent, D-GPM manages to overcome the above drawbacks. These prediction models are not perfect and behave better separately for different target genes. It is instructive to interpret these prediction models and explain the inherent relationship between the promoter methylation of the target genes and landmark genes according to our results. Although D-GPM is the best model for predicting most of the target genes, the three machine learning methods all have specific advantages when predicting some specific target genes. In future work, we will integrate multiple prediction models as an ensemble tool to ensure that it is suitable for predicting as many target genes as possible. In addition, we need to conduct a verification experiment to judge whether the conclusion drawn from the relationship between the promoter methylation of the target and landmark genes (such as *TDP1* and *CIAPIN1*) is sound and persuasive. Furthermore, after obtaining the ensemble prediction model, we will make the most of it to impute and revise methylation sites that are missing or have a low reliability in realistic methylation profiling data.

## 5. Conclusions

In summary, D-GPM acquires the least overall MAE and the highest PCC for the MBV-te among LR, RT, and SVM. For a gene-wise comparative analysis of D-GPM and the above three methods, D-GPM outperforms LR, RT, and SVM for predicting an overwhelming majority of the target genes, concerning the MAE and PCC. In addition, according to the model distribution of the least MAE and the highest PCC for the target genes, D-GPM predominates among the other models for predicting a majority of the target genes, laying a solid foundation for explaining the inherent relationship between the promoter methylation of target genes and landmark genes via interpreting results from these prediction models.

## Figures and Tables

**Table 1:** Detailed parameter configurations for D-GPM.

Parameters	
# of hidden layers	[1,2,3,4,5,6,7,8]
# of hidden units in each hidden layer	[1000,2000,3000,4000,5000,6000,7000,8000,9000]
Dropout rate	[0%, 5%,1 0%, 15%, 20%, 25%, 30%, 35%, 40%, 45%, 50%]
Momentum coefficient	0.5
Initial learning rate	5 × 10^−4^, 2 × 10^−4^, 1 × 10^−4^ or 8 × 10^−5^
Minimum learning rate	1.00 × 10^−5^
Learning rate decay factor	0.9
Learning scale	3.0
Mini-batch size	200
Training epoch	500
Weights’ initial range	$\left[-\frac{\sqrt{6}}{\sqrt{n_{i}+n_{o}}},\frac{\sqrt{6}}{\sqrt{n_{i}+n_{o}}}\right]$

**Table 2:** The mean absolute error (MAE)-based overall errors of linear regression (LR), the regression tree (RT), the support vector machine (SVM), and D-GPM, with partially different architectures (hidden layer: from 4 to 6, with step size 1; hidden unit: from 6000 to 8000, with step size 1000; dropout rate: from 10% to 20%, with step size 5%) for MBV-te. The numbers before “±” are the overall MAE for all the target genes. The numbers after “±” are the standard deviations of the predicted MAE over all the target genes. The best MAE performance of D-GPM is underlined. SD refers to the standard deviation.

Hidden Layers	Dropout Rate	Hidden Units		
		6000	7000	8000
4	10%	0.0332 ± 0.0253	0.0340 ± 0.0260	0.0343 ± 0.0263
4	15%	0.0333 ± 0.0253	0.0340 ± 0.0260	0.0344 ± 0.0264
4	20%	0.0336 ± 0.0255	0.0343 ± 0.0261	0.0344 ± 0.0262
5	10%	0.0344 ± 0.0264	0.0337 ± 0.0257	0.0346 ± 0.0264
5	15%	0.0343 ± 0.0260	0.0329 ± 0.0251	0.0343 ± 0.0261
5	20%	0.0350 ± 0.0267	0.0343 ± 0.0259	0.0347 ± 0.0265
6	10%	0.0341 ± 0.0259	0.0339 ± 0.0258	0.0339 ± 0.0258
6	15%	0.0339 ± 0.0259	0.0334 ± 0.0255	0.0331 ± 0.0253
6	20%	0.0356 ± 0.0269	0.0346 ± 0.0261	0.0351 ± 0.0265
**Linear regression**		0.0363 ± 0.0277		
**Support vector machine**		0.0350 ± 0.0258		
**Regression tree**		0.0454 ± 0.0363		

**Table 3:** The Pearson correlation coefficient (PCC) of LR, RT, SVM, and D-GPM, with partially different architectures (hidden layer: from 4 to 6, with step size 1; hidden unit: from 6000 to 8000, with step size 1000; dropout rate: from 10% to 20%, with step size 5%) for MBV-te. The numbers before “±” are the overall PCC for all the target genes. The numbers after “±” are the standard deviations of the PCC over all the target genes. The best PCC performance of D-GPM is underlined. SD refers to the standard deviation.

Hidden Layers	Dropout Rate	Hidden Units		
		6000	7000	8000
4	10%	0.8081 ± 0.0964	0.7972 ± 0.0976	0.8058 ± 0.0957
4	15%	0.8055 ± 0.0968	0.7936 ± 0.0989	0.8041 ± 0.0961
4	20%	0.8077 ± 0.0964	0.8032 ± 0.0964	0.7968 ± 0.0951
5	10%	0.7776 ± 0.1022	0.8032 ± 0.0984	0.7990 ± 0.0944
5	15%	0.7842 ± 0.1012	0.8186 ± 0.0940	0.7828 ± 0.1035
5	20%	0.7835 ± 0.1001	0.8135 ± 0.0943	0.7933 ± 0.0997
6	10%	0.7919 ± 0.0987	0.8007 ± 0.0961	0.8010 ± 0.0947
6	15%	0.7865 ± 0.1002	0.8086 ± 0.0923	0.8106 ± 0.0914
6	20%	0.7879 ± 0.1006	0.8082 ± 0.0925	0.7952 ± 0.0975
**Linear regression**		0.7846 ± 0.1069		
**Support vector machine**		0.7942 ± 0.1056		
**Regression tree**		0.6657 ± 0.1192		

## Supplementary Materials

The following are available online at https://www.mdpi.com/2073-4425/10/10/807/s1: The complete MAE and PCC evaluation of D-GPM armed with all the other architectures (hidden layer: from 1 to 8, with step size 1; hidden unit: from 1000 to 9000, with step size 1000; dropout rate: from 0% to 50%, with step size 5%) for the MBV-te is given in the file named “The complete evaluation.txt”.

## References

[1] Moore L.D., Le T., Fan G. (2013). DNA methylation and its basic function. Neuropsychopharmacol. Off. Publ. Am. Coll. Neuropsychopharmacol..

[2] Jones P.A. (2012). Functions of DNA methylation: Islands, start sites, gene bodies and beyond. Nat. Rev. Genet..

[3] Bird A. (2002). DNA methylation patterns and epigenetic memory. Genes Dev..

[4] Bestor T.H., Edwards J.R., Boulard M. (2015). Notes on the role of dynamic DNA methylation in mammalian development. Proc. Natl. Acad. Sci. USA.

[5] Huang Y.-W., Huang T.H.-M., Wang L.-S. (2010). Profiling DNA methylomes from microarray to genome-scale sequencing. Technol. Cancer Res. Treat..

[6] Laird P.W. (2010). Principles and challenges of genome-wide DNA methylation analysis. Nat. Rev. Genet..

[7] Plongthongkum N., Diep D.H., Zhang K. (2014). Advances in the profiling of DNA modifications: Cytosine methylation and beyond. Nat. Rev. Genet..

[8] Schwartzman O., Tanay A. (2015). Single-cell epigenomics: Techniques and emerging applications. Nat. Rev. Genet..

[9] Krygier M., Podolak-Popinigis J., Limon J., Sachadyn P., Stanislawska-Sachadyn A. (2016). A simple modification to improve the accuracy of methylation-sensitive restriction enzyme quantitative polymerase chain reaction. Anal. Biochem..

[10] Thu K.L., Vucic E.A., Kennett J.Y., Heryet C., Brown C.J., Lam W.L., Wilson I.M. (2009). Methylated DNA immunoprecipitation. J. Vis. Exp..

[11] Bibikova M., Le J., Barnes B., Saedinia-Melnyk S., Zhou L., Shen R., Gunderson K.L. (2009). Genome-wide DNA methylation profiling using Infinium(R) assay. Epigenomics.

[12] Li Q., Hermanson P.J., Springer N.M. (2018). Detection of DNA Methylation by Whole-Genome Bisulfite Sequencing. Methods Mol. Biol. (Clifton N.J.).

[13] Chen Y., Li Y., Narayan R., Subramanian A., Xie X. (2016). Gene expression inference with deep learning. Bioinformatics.

[14] Bansal M., Belcastro V., Ambesi-Impiombato A., di Bernardo D. (2007). How to infer gene networks from expression profiles. Mol. Syst. Biol..

[15] Edgar R., Domrachev M., Lash A.E. (2002). Gene Expression Omnibus: NCBI gene expression and hybridization array data repository. Nucleic Acids Res..

[16] Medvedeva Y.A., Khamis A.M., Kulakovskiy I.V., Ba-Alawi W., Bhuyan M.S.I., Kawaji H., Lassmann T., Harbers M., Forrest A.R., Bajic V.B. (2014). Effects of cytosine methylation on transcription factor binding sites. BMC Genom..

[17] Xiong H.Y., Alipanahi B., Lee L.J., Bretschneider H., Merico D., Yuen R.K., Hua Y., Gueroussov S., Najafabadi H.S., Hughes T.R. (2015). The human splicing code reveals new insights into the genetic determinants of disease. Science.

[18] Kelley D.R., Snoek J., Rinn J.L. (2016). Basset: Learning the regulatory code of the accessible genome with deep convolutional neural networks. Genome Res..

[19] Zhou J., Troyanskaya O.G. (2015). Predicting effects of noncoding variants with deep learning–based sequence model. Nat. Methods.

[20] Tomczak K., Czerwinska P., Wiznerowicz M. (2015). The Cancer Genome Atlas (TCGA): An immeasurable source of knowledge. Contemp. Oncol. (Poznan Poland).

[21] Touleimat N., Tost J. (2012). Complete pipeline for Infinium((R)) Human Methylation 450K BeadChip data processing using subset quantile normalization for accurate DNA methylation estimation. Epigenomics.

[22] Huang W.Y., Hsu S.D., Huang H.Y., Sun Y.M., Chou C.H., Weng S.L., Huang H.D. (2015). MethHC: A database of DNA methylation and gene expression in human cancer. Nucleic Acids Res..

[23] Srivastava N., Hinton G., Krizhevsky A., Sutskever I., Salakhutdinov R. (2014). Dropout: A simple way to prevent neural networks from overfitting. J. Mach. Learn. Res..

[24] Sutskever I., Martens J., Dahl G., Hinton G. On the importance of initialization and momentum in deep learning. Proceedings of the International Conference on Machine Learning.

[25] Goodfellow I.J., Wardefarley D., Lamblin P., Dumoulin V., Mirza M., Pascanu R., Bergstra J., Bastien F., Bengio Y. (2013). Pylearn2: A machine learning research library. arXiv.

[26] Bergstra J., Breuleux O., Bastien F., Lamblin P., Pascanu R., Desjardins G., Turian J., Warde-Farley D., Bengio Y. Theano: A CPU and GPU math expression compiler. Proceedings of the Python for Scientific Computing Conference (SciPy).

[27] Therneau T., Atkinson B., Ripley B. (2015). Package ‘rpart’. cran.ma.ic.ac.uk/web/packages/rpart/rpart.pdf.

[28] Steinwart I., Hush D., Scovel C. (2006). An Explicit Description of the Reproducing Kernel Hilbert Spaces of Gaussian RBF Kernels. IEEE Trans. Inf. Theory.

[29] Karatzoglou A., Smola A., Hornik K., Zeileis A. (2004). kernlab—An S4 Package for Kernel Methods in R. J. Stat. Softw..

[30] Hinton G.E., Srivastava N., Krizhevsky A., Sutskever I., Salakhutdinov R.R. (2012). Improving neural networks by preventing co-adaptation of feature detectors. Comput. Sci..

[31] Glorot X., Bengio Y. (2010). Understanding the difficulty of training deep feedforward neural networks. J. Mach. Learn. Res..

[32] Lewis R.J. An introduction to classification and regression tree (CART) analysis. Proceedings of the Annual Meeting of the Society for Academic Emergency Medicine.

[33] Park J.A., Jun K.R., Han S.H., Kim G.H., Yoo H.W., Hur Y.J. (2012). A novel mutation in the ABCD1 gene of a Korean boy diagnosed with X-linked adrenoleukodystrophy. Gene.

[34] Thodi G., Schulpis K.H., Dotsikas Y., Pavlides C., Molou E., Chatzidaki M., Triantafylli O., Loukas Y.L. (2016). Hawkinsinuria in two unrelated Greek newborns: Identification of a novel variant, biochemical findings and treatment. J. Pediatr. Endocrinol. Metab. JPEM.

[35] Wongprasert H., Somanunt S., De Filippo R., Picard J.Y., Pitukcheewanont P. (2013). A novel mutation of anti-Mullerian hormone gene in Persistent Mullerian Duct Syndrome presented with bilateral cryptorchidism: A case report. J. Pediatr. Urol..

[36] Ticho B.H. (2010). Iris transillumination defects associated with pallister-killian syndrome. J. Pediatr. Ophthalmol. Strabismus.

